# Pathology-validated structural and physiologic habitat imaging for differentiating radiation necrosis from tumor recurrence in brain metastases

**DOI:** 10.1007/s11060-026-05761-7

**Published:** 2026-08-28

**Authors:** Ji Eun Park, Guowen Shao, Shivani Baisiwala, Nakyoung Kim, Amelia Tan, Francesco Sanvito, Andrea Liang, Zexi Wang, Gianluca Nocera, Catalina Raymond, Viên Lam Le, Ho Sung Kim, Noriko Salamon, Whitney B. Pope, Won Kim, Benjamin M. Ellingson, Jingwen Yao

**Affiliations:** 1 Department of Radiology and Radiological Science, Johns Hopkins University, Baltimore, USA; 2 Department of Radiology, Asan Medical Center, Seoul, Korea, Republic of (South Korea); 3 UCLA Brain Tumor Imaging Laboratory (BTIL), Center for Computer Vision and Imaging Biomarkers, University of California, Los Angeles, CA USA; 4 Department of Bioengineering, Samueli School of Engineering, University of California, Los Angeles, CA USA; 5 Department of Neurosurgery, David Geffen School of Medicine, University of California, Los Angeles, CA USA; 6 Dynapex LLC, Seoul, South Korea; 7 Stanford University, Stanford, CA USA; 8 Department of Radiological Sciences, David Geffen School of Medicine, University of California, Los Angeles, CA USA; 9 Neuroscience Interdepartmental Program, University of California, Los Angeles, CA USA; 10 Department of Neurosurgery and Gamma Knife Radiosurgery, IRCCS Ospedale San Raffaele, Milan, Italy

**Keywords:** Stereotactic radiosurgery, Radiation necrosis, MRI, Tumor habitat analysis, Pathologic validation, Apparent diffusion coefficient, Cerebral blood volume, Voxel-wise clustering

## Abstract

**Purpose:**

Differentiating tumor recurrence from radiation necrosis (RN) after stereotactic radiosurgery (SRS) remains a major diagnostic challenge in brain metastasis. We aimed to validate established MRI-based tumor habitat analysis for distinguishing tumor from RN in an independent cohort with histopathological ground truth.

**Materials and methods:**

This retrospective study included 104 patients (104 lesions) with pathologically confirmed recurrent metastatic tumors (*n* = 68) or RN (*n* = 36) who underwent structural and physiologic MRI. Tumor habitats were generated using an established unsupervised clustering model applied to normalized T1-weighted enhanced, T2-weighted, apparent diffusion coefficient, and cerebral blood volume maps. Structural habitats (enhancing tissue, solid low-enhancing, nonviable) and physiologic habitats (hypervascular, hypovascular cellular, nonviable) were quantified as absolute volumes and volume fractions. Logistic regression and receiver operating characteristics analysis evaluated the ability to differentiate tumor and RN. Composite habitat scores integrating structural and physiologic habitats were also developed.

**Results:**

Recurrent metastatic tumors showed higher contrast-enhancing volume (*P* = .006), higher solid low-enhancing habitat volume (*P* = .029) and fraction (*P* = .04), higher hypervascular habitat volume (*P* = .02) and fraction (*P* = .03), and lower nonviable tissue habitat fractions on structural (*P* = .003) and physiologic MRI (*P* = .015), compared with RN. The combined structural and physiologic MRI habitat score showed the highest diagnostic performance (AUC, 0.80; 95% CI: 0.71–0.87; sensitivity, 89.7%; specificity, 58.3%).

**Conclusion:**

MRI-based tumor habitat analysis provides a pathology-validated approach to distinguish tumor recurrence from radiation necrosis in patients with prior radiation therapy.

**Supplementary Information:**

The online version contains supplementary material available at https://doi.org/10.1007/s11060-026-05761-7.

## Summary statement

In patients with brain metastases and prior radiation therapy, histologically validated voxel-based MRI habitat analysis improves differentiation of tumor recurrence from radiation necrosis after stereotactic radiosurgery, with solid low-enhancing and hypervascular regions indicating viable tumor.

**Supplementary Information:**

The online version contains supplementary material available at https://doi.org/10.1007/s11060-026-05761-7.

## Key results

In patients with prior radiation therapy, structural and physiologic tumor habitat analysis distinguishes tumor from radiation necrosis in BM in an independent cohort with histological ground truth.

A high fraction of solid low-enhancing or hypervascular habitat and a low fraction of nonviable tissue habitat favored tumor recurrence in the post-radiation setting.

A composite habitat score combining structural and physiologic MRI achieved the highest diagnostic performance in the prior-radiation cohort.

**Supplementary Information:**

The online version contains supplementary material available at https://doi.org/10.1007/s11060-026-05761-7.

## Introduction

Increase in size of existing contrast enhancing mass after radiation therapy, especially stereotactic radiosurgery (SRS) complicates response assessment in patients with brain metastasis (BM), as it raises the challenge of differentiating radiation necrosis from tumor recurrence [1, 2]. However, no definite imaging biomarker has yet demonstrated a reliable diagnostic performance with sufficient accuracy [3–5]. Physiologic MRI techniques including diffusion-weighted imaging (DWI) with apparent diffusion coefficient (ADC) [6–8] and dynamic susceptibility contrast (DSC) with cerebral blood volume (CBV) [6, 9–11] have shown potential, but their ability to distinguish two entities considerably varied [4]. On the other hand, structural MRI using ratio of low T2 signal on T1 enhancement ratio has suggested diagnostic value [12], but this metric has shown poor reproducibility and sensitivity [12, 13].

The lack of a reliable imaging marker arises partly because brain metastases exhibit diverse tumor microenvironment, with variable cellularity and vascularity according to the primary cancer [14]. Moreover, radiation necrosis and tumor recurrence may coexist in SRS-treated tissue, with intermixed viable tumor, capillary damage, ischemia, and chemotherapy-related damage [13, 15–17]. Combination of different imaging biomarkers of ADC, CBV, and structural MRI enables to measure intratumoral heterogeneity [18]. Tumor habitat analysis, which captures intratumoral heterogeneity by identifying similar voxels and subregions with shared biological features based on unsupervised clustering, may provide additional diagnostic value [18–20]. Recent studies have reported that a high volume fraction of solid, less-enhancing habitat, defined by low T2 and low T1 contrast-enhancement on structural MRI [21], and an increase of hypovascular cellular habitat, defined by low ADC and low CBV on physiologic MRI immediate post-SRS [22], was associated with future recurrence in brain metastasis. Such habitat analysis may improve reproducibility in identifying viable tumor versus treatment effect compared to using a single parametric map with fixed-cut-off threshold, as multiparametric clustering may identify more biologically plausible tumor subregions than relying on arbitrary individual voxel significance [23, 24]. Also, spatial parcellation of habitats assists in assessing residual tumor burden in post-treatment metastatic tumors, and in determining the target for further SRS.

Nevertheless, prior studies have been limited by small sample sizes and sparse pathological correlation, making validation of habitat analysis difficult. We hypothesized that applying tumor habitat analysis to an independent dataset of pathologically confirmed recurrent brain metastases and radiation necrosis, using structural MRI, diffusion, and perfusion MRI, would enable validation of established habitat imaging approaches. The purpose of our study was to evaluate the performance of established tumor habitat imaging techniques using structural and physiologic MRI in distinguishing tumor from RN in an independent dataset with histopathological ground truth.

## Materials and methods

### Patient population

**Fig. 1:**
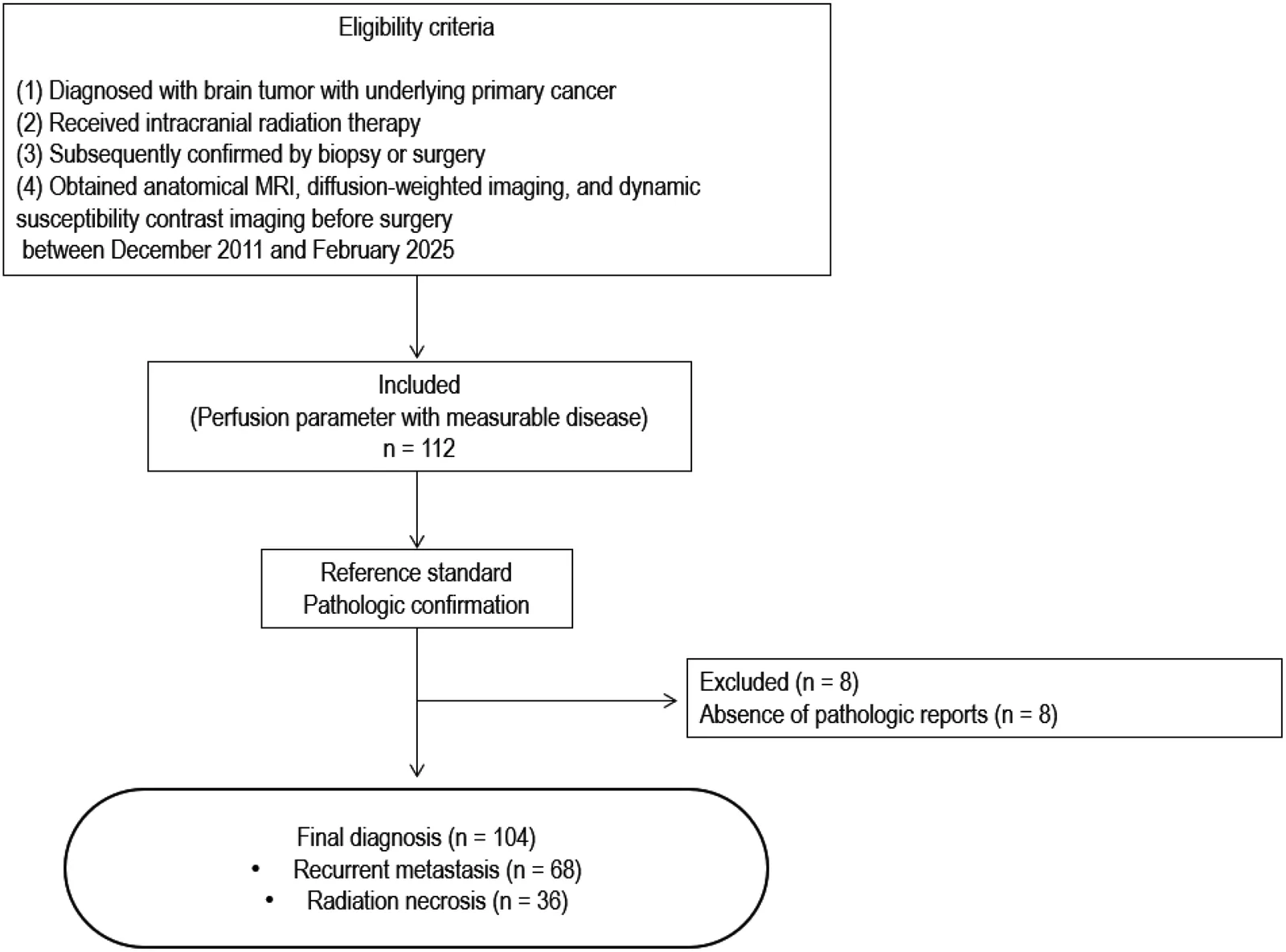
Inclusion process of patient cohort

This retrospective study was conducted in accordance with the Declaration of Helsinki and all relevant guidelines and regulations and was approved by the Ethics Committee and Institutional Review Board at University of California, Los Angeles (approval no. 20–000170 and 23-001415). Informed patient consent was obtained from all participants prior to the collection and analysis of data. Eligible patients had a diagnosis of brain tumor with underlying primary cancer, history of intracranial radiation therapy for brain metastasis, and subsequently histopathologic confirmation by biopsy or surgery between December 2011 and February 2025. Patients were drawn from cohorts in the two protocols. Inclusion criteria were: (a) availability of anatomic MRI, diffusion-weighted imaging (DWI), and dynamic susceptibility contrast (DSC) perfusion MRI prior to surgery or biopsy; (b) presence of a contrast-enhancing mass at least 1 × 1 × 1 cm; and (c) underwent surgery or biopsy after imaging and histopathologic confirmation of recurrent/progressive brain metastasis or radiation necrosis is available. From the aggregated dataset of 112 subjects, 8 were excluded due to absence of pathology reports. The final study population consisted of 104 patients (Fig. 1). The cohort included 68 patients with progressed/recurrent metastatic tumor and 36 patients with radiation necrosis.

### Histopathologic validation

Among 104 patients, 29 patients underwent biopsy and 75 patients underwent resection. Pathologic classification was based on review of the original clinical pathology reports. The pathologists were blinded to the habitat analysis at the time of review. The pathologic diagnosis was assigned to either tumor or treatment-related change by reviewing histopathologic records. Treatment effect was assigned when the records had only described lesion as “treatment effect”, “treatment-related changes”, “extensive necrosis with histiocytic infiltrate and gliosis’’, “gliosis”, or “necrosis/calcification”. Tumor was assigned when the records had “metastatic carcinoma” without other description. Mixed was assigned for 9 cases when the description of “consistent with metastatic carcinoma” and “extensive reactive change” or “extensive necrosis” coexisted. Considering that the presence of active tumor tissue warrants further treatment in clinical decision making, these mixed pathology lesions were assigned to the tumor group for analyses.

### MRI protocol

Anatomic and physiologic MRI were performed on 1.5T or 3T scanners. Anatomic sequences included 3D post-contrast T1-weighted imaging (CE-T1WI), T2-weighted imaging (T2WI), and fluid-attenuated inversion recovery (FLAIR) imaging, while physiological sequences included DWI and DSC imaging [25, 26]. Detailed sequence parameter ranges are included in Supplementary Table 1.

### Image data

#### Mask segmentation

Three volumes of interest (VOIs) were segmented using deep-learning-based segmentation algorithms [27], including the contrast-enhancing lesions (CEL) on post-contrast T1-weighted image, FLAIR hyperintensity lesions, and the entire white matter segmentation. Manual corrections were performed when deemed necessary. Voxels within the CEL segmentation were used for habitat analysis.

#### Preprocessing

Apparent diffusion coefficient (ADC) maps were calculated from b-values of 0 and 1000 s/mm² using a two-point signal decay estimate. DSC perfusion MRI was motion-corrected using the FSL function, MCFLIRT (https://fsl.fmrib.ox.ac.uk/fsl/fslwiki/MCFLIRT) [28], and relative cerebral blood volume maps (rCBV) were generated after correction for bi-directional contrast leakage [29, 30]. Then, normal appearing white matter (NAWM) was calculated by subtracting the CELs and FLAIR hyperintensity lesions from the entire white matter segmentation. For physiological MRI (ADC and CBV), the signal normalization was performed for CBV maps only. The normalized relative cerebral blood volume (nCBV) maps were generated by dividing rCBV by the mean NAWM rCBV value. For anatomic MRI (T2WI and CE-T1WI), the normalization of signal intensity was performed by z-score normalization of the signal intensity of each voxel by the mean and standard deviation of the NAWM signal intensity.

#### Tumor habitat analysis

FLAIR, nCBV, and ADC images were co-registered and resampled to isotropic CE-T1WI using rigid transformation (SPM12; Wellcome Trust Centre for Neuroimaging, London, UK). The voxel size for each imaging is 1 × 1 × 1 mm. For registration and tumor habitat analysis, we used a pre-built habitat analysis was applied from a research software (Dynapex LLC). The thresholds for voxel cluster assignment, k-means centroids, and clustering decision boundaries were derived from the clustering model for brain metastasis after radiation therapy built on prior data [21, 22]. The previous model-development dataset consisted of 103 stereotactic radiosurgery–treated lesions (37 tumor recurrence and 66 radiation necrosis) from 83 patients of brain metastasis from solid tumors (lung cancer, 48 patients; breast cancer 12 patients, others 23 patients) obtained multiparametric MRI between 2014 and 2020 in a tertiary hospital in Asia. The present cohort was independently collected at a tertiary hospital in the United States between December 2011 and February 2025. There was no patient, lesion, or institutional overlap between the two datasets, and no scanner hardware was shared between the institutions. The same processing and normalization method were applied in the original dataset, followed by unsupervised k-means clustering of voxel values into three anatomical habitats based on normalized CE-T1WI and T2WI, and three physiological habitats based on ADC and nCBV. The previously established k-means centroids and clustering boundaries used to assign voxels to structural and physiologic habitats were applied to the current MRI data without retraining, refitting, or modification.Structural MRI habitats were defined as: (a) enhancing tissue (high CE-T1 signal intensity irrespective of T2 signal); (b) solid low-enhancing tissue (low T2 and CE-T1 signal); and (c) nonviable tissue (high T2 and low CE-T1 signal).

Physiologic MRI habitats were defined as: (a) hypervascular (high CBV); (b) hypovascular cellular (low ADC, low CBV); and (c) nonviable tissue (high ADC, low CBV). In this study, high and low CBV refer to high and low normalized CBV (nCBV), respectively, but are denoted simply as CBV hereafter.

The establishment of tumor habitat is demonstrated in Supplementary Fig. 1.

### Statistical analysis

All results were reported as mean ± standard deviation for continuous variables and as frequencies (percentages) for categorical variables. Demographic characteristics between metastatic tumor and radiation necrosis groups were compared using the χ² test or Fisher exact test for categorical variables and the Student’s *t*-test for continuous variables.

Tumor habitat measures were compared across cancer types using one-way analysis of variance or the Kruskal–Wallis test, according to data distribution. Associations between imaging characteristics, including CEL volume and tumor habitats, and pathology were assessed with univariable logistic regression, with calculation of odds ratios (ORs) and *P* values. Tumor habitats were analyzed both as absolute volume (mL) and as volume fraction (%). Multivariable analysis was not performed due to small number of subjects.

Diagnostic thresholds for habitat absolute volume and volume fractions were selected in the current post-radiation cohort. The composite habitat score was developed post hoc using these current-cohort thresholds. Each significant variable was assigned a discrete score of 1 if greater than its threshold and 0 if less. For example, if two habitats associated with tumor exceeded their thresholds, the habitat risk score was 2. Structural MRI–based and physiologic MRI–based habitat scores were calculated for each lesion. The sum habitat scores were calculated by adding structural and physiologic MRI-based habitat scores. The sum habitat scores range from 0 to 4.

Diagnostic performance of the imaging characteristics and the calculated habitat scores for differentiating metastatic tumor from radiation necrosis was evaluated with the area under the receiver operating characteristic curve (ROC AUC) with 95% confidence interval. The comparison of ROC was performed with DeLong’s method.

All statistical analyses were performed using the R Statistical Package (version 4.4.1, Institute for Statistics and Mathematics, Vienna, Austria, http://www.R-project.org). A *P* value less than 0.05 was considered to indicate statistical significance.

## Results

### Patient population

The characteristics of the patients and lesions are summarized in Table 1. The primary analysis cohort included 104 patients with prior radiation therapy: 68 patients with metastatic tumor and 36 patients with radiation necrosis. Most patients received stereotactic radiosurgery (55 patients [80.9%] with tumor and 31 patients [86.1%] with radiation necrosis, respectively). There was no significant difference in age or sex distribution between the metastatic tumor and radiation necrosis groups.

**Table 1:** Characteristics of the patients and lesions

	Metastatic Tumor	Radiation necrosis	*P* value
Number of patients	68	36	
Age (years)	60.3± 14.9	62.7 ± 12.6	*0.263*
Men	23 (33.8)	16 (44.4)	*0.289*
Primary cancer			.*582*
Lung (non-small cell lung cancer)	20 (29.4)	14 (38.9)	
Breast	21 (30.9)	10 (27.7)	
Melanoma	11 (16.2)	6 (16.7)	
Colorectal cancer	4 (5.9)	0	
Renal cell cancer	0	0	
Others	12 (17.6)	6 (16.7)	
Lesion location			0.317
Frontal	29 (42.6)	14 (38.9)	
Parietal	11 (16.2)	11 (30.6)	
Temporal	12 (17.6)	5 (13.8)	
Occipital	12 (17.6)	3 (8.3)	
Thalamus	0 (0)	1 (2.8)	
Infratentorial	4 (5.9)	2 (5.6)	
Prior local treatments			
S/P Radiation	68 (36.2)	36 (100)	
Stereotactic radiosurgery (SRS)	55 (80.9)	31 (86.1)	0.797
Intensity-modulated radiation therapy (IMRT)	5 (7.3)	2 (5.6)	
Whole-brain radiation therapy (WBRT)	8 (11.8)	3 (8.3)	

Results are reported as the number (percent) or as mean ± standard deviation

Among the 68 recurrent metastatic tumors, 20 were non–small cell lung cancer (NSCLC), 21 breast cancer, 11 melanoma, 4 colorectal carcinoma, and 12 others (4 carcinomas, 2 sarcomas, 2 small cell lung cancer, 2 adenocarcinoma, and 2 neuroendocrine tumors). Among the radiation necrosis cases, 14 were NSCLC, 10 breast cancer, 6 melanoma, and 6 others (2 carcinomas, 2 sarcomas, and 2 germ cell tumors). There was no significant difference in tumor pathology between the two groups.

### Tumor habitat analysis

The differences in structural and physiologic MRI parameters between brain metastases and radiation necrosis and their univariable logistic regression analysis results are summarized in Table 2. Recurrent tumors showed higher contrast-enhancing volume (12.2 ± 11.1 vs. 6.0 ± 6.6 mL, *P* = .006), higher enhancing habitat volume (3.6 ± 5.1 vs. 1.6 ± 2.4 mL, *P* = .041), solid low-enhancing habitat volume (2.5 ± 3.7 vs. 0.8 ± 1.7 mL, *P* = .029), higher solid low-enhancing habitat fraction (19.8 ± 22.3 vs. 11.2 ± 11.2%, *P* = .04), and lower nonviable tissue habitat fraction on structural MRI (50.2 ± 27.9 vs. 67.9 ± 23.2%, *P* = .003).

**Table 2:** Difference in structural and physiologic MRI habitats between metastatic tumor and radiation necrosis and association for differentiating recurrent tumor from radiation necrosis in patients with history with radiation therapy

Imaging Parameters	Recurrent Tumor (*n* = 68)	Radiation Necrosis (*n* = 36)	Odds ratio	*P*	AUC	Sensitivity	Specificity	Threshold
Enhancing volume (mean (SD))	12.2 ± 11.1	6.0 ± 6.6	1.01 (1.00-1.02)	**0.006**	0.70 (0.61–0.79)	75.0	69.4	> 4.6
Structural MRI								
Volume (mL)								
Enhancing habitat	3.6 ± 5.1	1.6 ± 2.4	1.02 (1.00-1.04)	**0.041**	0.67 (0.58–0.76)	69.1	66.7	> 0.6
Solid low-enhancing habitat	2.5 ± 3.7	0.8 ± 1.7	1.03 (1.01–1.07)	**0.029**	0.68 (0.58–0.77)	58.8	86.1	> 0.8
Nonviable tissue habitat	6.1 ± 7.8	3.6 ± 4.6	1.01 (0.90–1.02)	0.10				
Volume fraction (%)								
% Enhancing habitat	30.1 ± 25.3	20.9 ± 21.6	1.02 (0.99–1.04)	0.07				
% Solid low-enhancing habitat	19.8 ± 22.3	11.2 ± 11.2	1.03 (1.00-1.06)	**0.04**	0.58 (0.49–0.68)	32.3	86.1	> 24.4%
% Nonviable tissue habitat	50.2 ± 27.9	67.9 ± 23.2	0.97 (0.96–0.99)	**0.003**	0.68 (0.58–0.77)	63.2	75.0	≤ 57.1%
Physiologic MRI								
Volume (mL)								
Hypervascular habitat	1.7 ± 3.5	0.2 ± 0.9	1.09 (1.03–1.20)	**0.02**	0.80 (0.71–0.87)	80.9	69.4	> 0.3
Hypovascular cellular habitat	8.0 ± 8.2	3.9 ± 4.8	1.01 (1.00-1.02)	**0.009**	0.70 (0.60–0.79)	70.6	72.2	> 3.2
Nonviable tissue habitat	2.4 ± 3.8	1.9 ± 2.2	1.00 (0.99–1.02)	0.428				
Volume fraction (%)								
% Hypervascular habitat	11.8 ± 19.1	3.1 ± 9.4	1.07 (1.02–1.15)	**0.03**	0.78 (0.69–0.86)	89.7	58.3	> 1.3%
% Hypovascular cellular habitat	66.8 ± 21.8	65.0 ± 25.7	1.00 (0.98–1.02)	0.707				
% Nonviable tissue habitat	21.4 ± 17.1	31.9 ± 23.9	0.97 (0.95–0.99)	**0.015**	0.63 (0.53–0.72)	61.8	61.1	≤ 21.1%

Note: Odd ratios reported here indicate the relative change in odds that a 1-unit (mL or %) increase in each imaging parameter incurs. Numbers in parentheses are 95% confidence interval

On physiologic MRI, both hypervascular habitat and hypovascular cellular habitat volumes were larger in metastatic tumors compared with radiation necrosis (hypervascular: 1.7 ± 3.5 vs. 0.2 ± 0.9 mL, *P* = .02; hypovascular: 8.0 ± 8.2 mL vs. 3.9 ± 4.8 mL, *P* = .009). The volume fraction of hypervascular habitat was also greater in metastatic tumor (11.8 ± 19.1 vs. 3.1 ± 9.4%, *P* = .03), while nonviable tissue habitat was smaller (21.4 ± 17.1% vs. 31.0 ± 24.0%, *P* = .015). On the other hand, volume fraction of hypovascular cellular habitat was similar between two entities (*P* = .707). Supplementary Fig. 2 demonstrates the difference in volume fraction between two groups.

## Differentiation of tumor recurrence from radiation necrosis using tumor habitat analysis

**Fig. 2:**
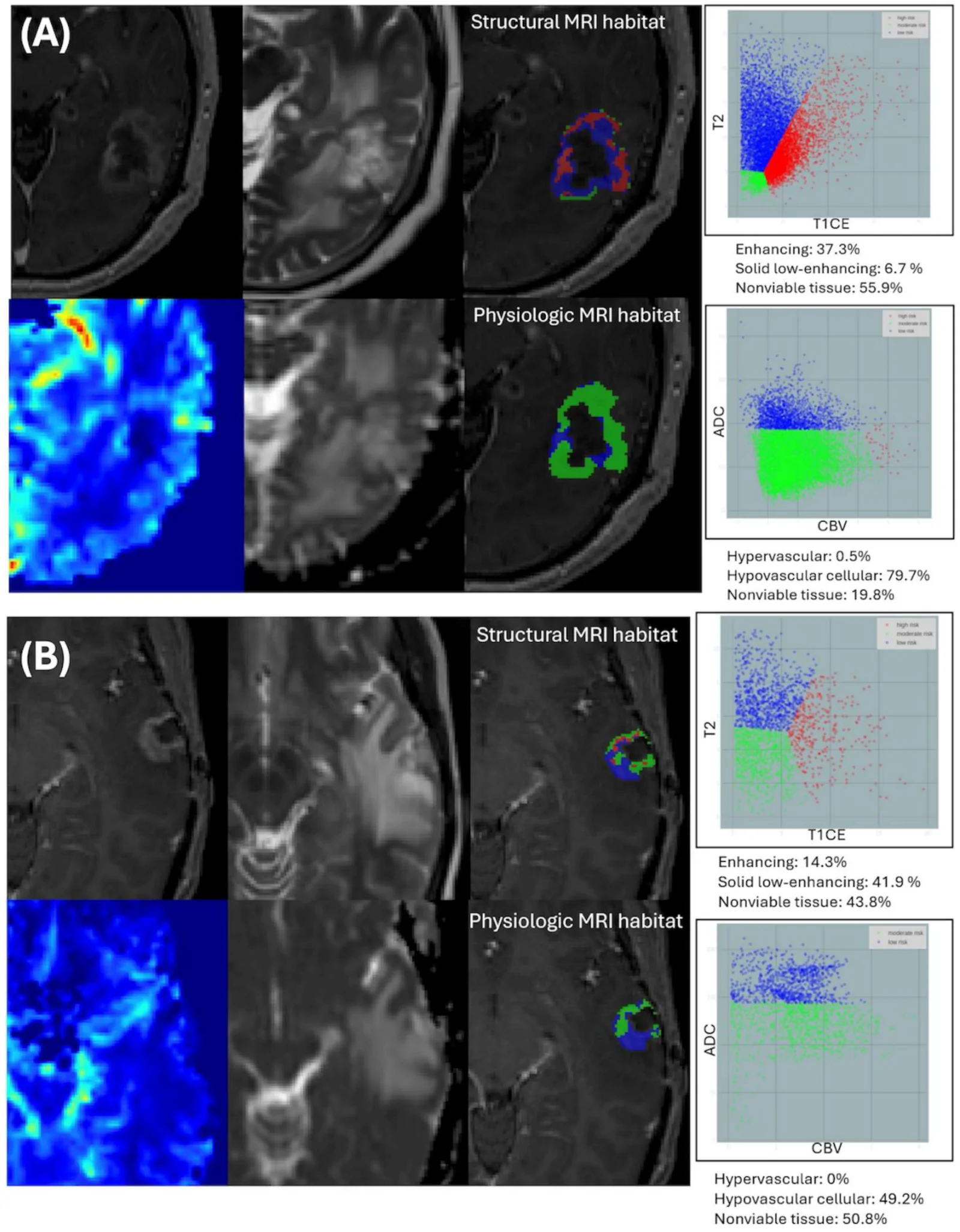
Comparison of structural MRI and physiologic MRI-based tumor habitat analysis in patients with radiation necrosis. (**A**) A 67-year-old female with lung cancer (adenocarcinoma) treated with SRS for the left temporal lobe lesion. On structural MRI, nonviable tissue habitat was dominant (55.9%) and solid low-enhancing habitat was minimal (6.7%). On physiologic MRI, minimal hypervascular habitat was noted (0.5%) with more than 16.5% of nonviable tissue habitat (19.8%). (**B**) A 71-year-old female with breast cancer (invasive ductal carcinoma) treated with SRS for the left temporal lobe lesion. On structural MRI, nonviable tissue habitat was 43.8% with less than 76.3% of solid low-enhancing habitat (41.9%). On physiologic MRI, no hypervascular habitat (0%) with prominent nonviable tissue habitat (50.8%)

**Fig. 3:**
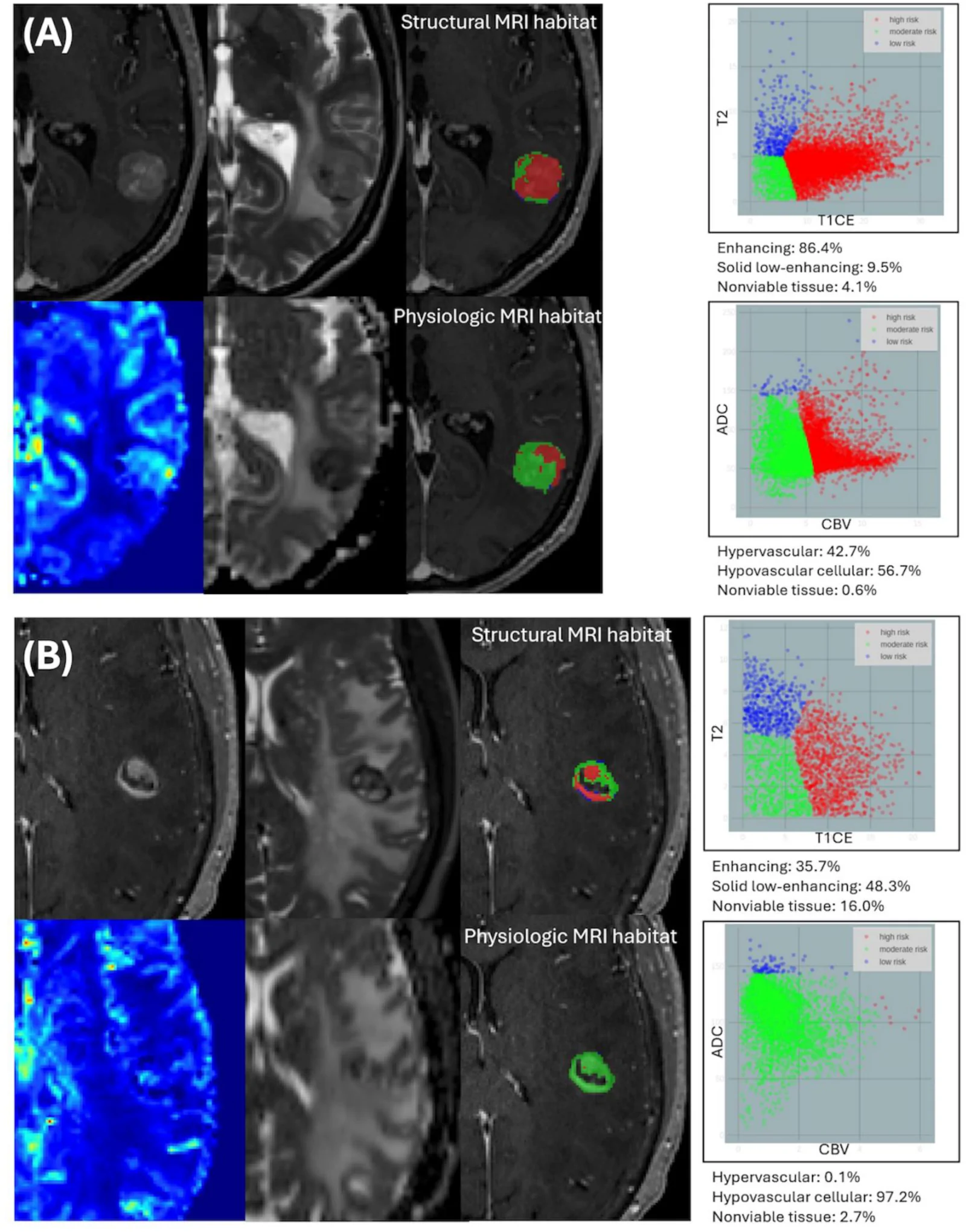
Comparison of structural MRI and physiologic MRI-based tumor habitat analysis in patients with tumor recurrence. (**A**) A 58-year-old female with breast cancer treated with SRS for the left temporal lobe lesion. On structural MRI, nonviable tissue habitat was scanty (4.1%) and enhancing habitat was predominant (86.4%). On physiologic MRI, prominent hypervascular habitat (42.7%) and scanty amount of nonviable tissue habitat (0.6%) is noted. (**B**) A 71-year-old female with melanoma treated with SRS for left temporal lobe lesion. On nonviable tissue was less than 16.5% on structural MRI (16.0%) and 10.1% on physiologic MRI (2.7%), respectively

Univariable logistic regression analysis to differentiate tumor recurrence from radiation necrosis is shown in Table 2. On structural MRI, higher volume fraction of solid low–enhancing habitat (OR, 1.03; 95% CI: 1.01–1.06; *P* = .04) with threshold > 24.4% and lower volume fraction of nonviable tissue habitat (OR, 0.97; 95% CI: 0.96–0.99; *P* = .003) with threshold ≤ 57.1% were associated with tumor recurrence. On physiologic MRI, higher volume fraction of hypervascular habitat (OR, 1.07; 95% CI: 1.02–1.15; *P* = .03) with threshold > 1.3% and lower volume fraction of nonviable tissue habitat (OR, 0.97; 95% CI: 0.95–0.99; *P* = .015) with threshold ≤ 21.1% were associated with tumor recurrence. Representative cases of radiation necrosis and metastatic tumor are shown in Figs. 2 and 3, respectively.

### Diagnostic performance from combined tumor habitat score

The diagnostic thresholds for the volume fractions of solid low-enhancing habitat (> 24.4%), structural nonviable tissue habitat (≤ 57.1%), hypervascular habitat (> 1.3%), and physiologic nonviable tissue habitat (≤ 21.1%) were selected to construct composite habitat scores. Using these thresholds, composite habitat scores were calculated for each lesion. The diagnostic performances in the primary cohort of patients with radiation therapy and all patients are shown in Table 3. The physiologic MRI habitat score showed slightly higher performance (AUC 0.75, 95% CI: 0.65–0.83) than the structural MRI habitat score (AUC 0.69, 95% CI: 0.59–0.78). The combined structural and physiologic MRI habitat score showed the best performance, with an AUC of 0.80 (95% CI: 0.71–0.87), sensitivity of 89.7%, and specificity of 58.3%, which was significantly better than the structural MRI score alone (AUC, 0.69 [95% CI: 0.59–0.78], DeLong’s method, *P* < .001).

**Table 3:** Diagnostic performance of MRI habitat scores for differentiating metastatic tumor from radiation necrosis

	AUC (95% CI)	Sensitivity	Specificity	*P* value
Structural MRI habitat score	0.69 (0.59–0.78)	67.7%	66.7%	< 0.001
Physiologic MRI habitat score	0.75 (0.65–0.83)	66.2%	80.6%	0.161
Combination of structural and physiologic MRI habitat score	0.80 (0.71–0.87)	89.7%	58.3%	Ref

AUC = area under the receiver-operating-characteristics curve, 95% CI = 95% confidence interval, PPV = positive predictive value, NPV = negative predictive value

## Discussion

In this study, we demonstrated that both structural MRI-based habitat and physiologic MRI-based habitat analysis are useful for differentiating brain metastases from radiation necrosis, validated against pathologic findings. A previously developed and fixed habitat-assignment model using dataset from prior studies [21, 22] was successfully applied to an independent pathology-confirmed cohort without refitting, supporting the generalizability of the approach. The diagnostic thresholds and composite habitat score were derived in the present cohort with pathologic validation, which requires future validation in a larger cohort.

A large lesion with tumor recurrence may have a substantial absolute volume of each habitat simply because the lesion itself is larger. Expressing each habitat as a volume fraction inherently accounts for lesion size by representing each habitat as a proportion of the entire enhancing lesion. Using T1- and T2-weighted images, the volume fraction of solid low-enhancing and nonviable tissue habitats provided diagnostic value. Specifically, > 24.4% of solid low-enhancing habitat or ≤ 57.1% of nonviable tissue habitat indicated tumor recurrence. Using ADC and CBV maps, the volume fractions of hypervascular and nonviable tissue habitats were helpful. In particular, > 1.3% of hypervascular habitat and ≤ 21.1% of nonviable tissue habitat were diagnostic of recurrence. When combined into a habitat score integrating both structural and physiologic MRI, the diagnostic performance reached an AUC of 0.80. These results suggest that tumor habitat analysis with pathological correlation can serve as a non-invasive tool for distinguishing recurrence from radiation necrosis on cross-sectional MRI in patients with treated brain metastases.

Our findings are consistent with previous imaging biomarker research showing that combining imaging parameters helps delineate the tumor microenvironment into biologically distinct subregions. Using structural MRI, prior studies of T1/T2 mismatch (non-matching lesion boundaries on T2-weighted and contrast-enhanced T1-weighted imaging) reported sensitivity of 15%–83.3% and specificity of 91.1%–100% for radiation necrosis [12, 31]. However, reproducibility was limited by manual segmentation and lack of signal intensity normalization [32, 33]. In our study, we found that solid low-enhancing habitat (low T2/ low T1CE) and nonviable tissue habitat (high T2/low T1CE), which corresponds to T1/T2 mismatch, also showed high diagnostic performance, consistent with previous cross-sectional habitat studies [21, 22]. Automated deep-learning–based segmentation was applied to the entire contrast-enhancing lesion and a white-matter intensity normalization method was implemented across different MRI protocols. This approach improves reproducibility, enables quantitative analysis, and promotes standardization across sites.

The physiologic MRI parameters become the most significant variables for tumor recurrence from radiation necrosis. Based on single habitat, volume fraction of hypervascular habitat reflecting high rCBV portion in the enhancing lesion showed highest diagnostic performance with AUC 0.82 with 3.3% cut-off of volume fraction. These results are consistent with prior reports that high rCBV reflects tumor recurrence [6, 9–11], although prior studies reported variable cutoff values ranging from 1.52 to 2.1 and lacked full validation against pathologic data. On meta-analysis of MR perfusion, overall diagnostic performance using single parameter of rCBV between radiation necrosis and tumor recurrence reported as AUC 0.87 with pooled sensitivity 81.6% and pooled specificity 80.6% from 10 different studies with 96 post-SRS metastases [34]. The diagnostic performance in our study is comparable to prior studies. Moreover, habitat analysis has strength that includes the volume fraction of high rCBV by voxel-based tumor clustering that reflects both high vascularity and the proportion of it within the enhancing lesion; this will be helpful to detect the location of tumor recurrence and the site of future targeted therapy including re-SRS.

For hypovascular cellular habitat, a higher volume was associated with tumor recurrence, but its volume fraction was not indicative of tumor recurrence. The reason why the volume fraction of hypovascular cellular habitat was non diagnostic can be explained in several ways. First, baseline ADC values varied by histology; for example, the ADC values of breast cancer metastases differ significantly according to receptor mutations [35], and the ADC values of lung cancer metastases differ significantly according to whether the lesions are metastases of small-cell lung cancer, adenocarcinoma, or squamous cell carcinoma [36]. Radiation necrosis itself shows decreased ADC due to radiation-induced ischemia, which complicates diagnosis [3, 37]. A previous habitat analysis [22] showed that an increase in hypovascular cellular habitat in longitudinal analysis was associated with future recurrence and the site of recurrence. Therefore, volume change can become a helpful biomarker, especially hypovascular cellular habitat using physiologic MRI on longitudinal analysis.

Although the combined habitat score showed relatively high sensitivity in the post-radiation cohort, its specificity was modest at 58.3%. Consequently, the score should not be used as a stand-alone determinant of recurrent tumor or as the sole basis for invasive treatment decisions. Rather, habitat analysis may provide complementary quantitative information when interpreted together with serial imaging, clinical findings, treatment history, and multidisciplinary assessment. Independent validation and comparison with expert qualitative interpretation are required before clinical application.

MRI-based tumor habitat analysis can be adjunct tool similar to MR spectroscopy or PET. Using MR spectroscopy, Cho/Cr ratio or Cho/NAA ratio showed sensitivity and specificity reaching up to 91% and 95%, though with substantial heterogeneity across studies in a meta-analysis [38]. Compared with MR spectroscopy, MRI-based tumor habitat analysis enables the depiction of intratumoral heterogeneity and spatial localization. Similarly, a recent meta-analysis reported that amino acid PET with ^11^C‐MET, ^18^F‐FET and ^18^F‐FDOPA showed a pooled sensitivity and specificity of 82% and 84%, respectively [39]. Compared with amino acid PET, MRI-based tumor habitat analysis does not require additional radiation exposure or radiotracer administration and provides substantially higher spatial resolution. Furthermore, multiple structural, diffusion, and perfusion imaging biomarkers can be integrated within a single MRI examination, eliminating the need for a separate imaging session. Because MRI habitat analysis provides spatially resolved information derived from conventional structural, diffusion, and perfusion MRI sequences that are often already included in routine brain tumor imaging protocols, it may serve as a complementary imaging biomarker rather than a replacement for amino acid PET or MR spectroscopy.

This study has several limitations. First, although the diagnostic performance was promising, it was not high and multivariable analysis was not performed due to small sample size. Nevertheless, biological validation was achieved in all patients and the number of patients is largest until now. Second, this study evaluated a previously developed, fixed MRI habitat-assignment model in an independent pathology-confirmed cohort of previously irradiated brain metastases. Whether MRI habitat analysis provides incremental diagnostic value beyond the current neuroradiologist workflow remains unknown. The added value of MRI habitat analysis to existing workflow of neuroradiologist’s decision needs to be studied as a future design to demonstrate real clinical value. Third, although habitat volume fractions were used instead of absolute habitat volumes to reduce the influence of total lesion size, normalization by lesion volume does not completely eliminate size-related effects. In very small lesions, the limited voxel count may amplify measurement variability and reduce the robustness of volume-fraction estimates. Therefore, the proposed thresholds should be interpreted cautiously when applied to small enhancing lesions until further validation is available. Fourth, normalization to NAWM was the primary strategy used to mitigate variability related to scanner type, field strength, and acquisition protocol. However, this approach cannot fully eliminate interscanner and interprotocol variability. Further image-harmonization strategies and sensitivity analyses should be considered in a larger cohort. Finally, diagnostic performance was assessed across all metastases without stratification by pathology. Future large-scale studies incorporating pathology-specific analyses are needed to establish individualized cut-offs according to tumor histopathology.

In conclusion, tumor habitat analysis integrating structural and physiologic MRI offers a reproducible method to distinguish tumor recurrence from radiation necrosis in brain metastases. This may be helpful in monitoring patients after SRS and guiding future therapy by localizing the site of recurrence.

## Supplementary Information

Below is the link to the electronic supplementary material.

Supplementary Material 1

## Data Availability

No datasets were generated or analysed during the current study.

## Abbreviations

SRS: Stereotactic radiosurgery
RN: Radiation necrosis
ADC: Apparent diffusion coefficient
CBV: Cerebral blood volume
RANO-BM: Response Assessment in Neuro-Oncology criteria for brain metastases
HR: Hazard ratio
