# Supplementary material for: Pathology-validated structural and physiologic habitat imaging for differentiating radiation necrosis from tumor recurrence in brain metastases
Source: J Neurooncol. 2026 Aug 28;179(2):61. doi: 10.1007/s11060-026-05761-7 (PMC13525006; doi:10.1007/s11060-026-05761-7)
Supplement: Supplementary file 1 [file 11060_2026_5761_MOESM1_ESM.docx]

**Supplementary Table 1. Sequence acquisition parameter range of T1WI, T2WI, DWI, and DSC**

| Imaging Parameters | T1WI | T2WI | DWI | DSC |
| --- | --- | --- | --- | --- |
| Echo Time (msec) | 2.5 – 4.2 | 62 – 412 | 64 – 108 | 17 – 54 |
| Repetition Time (msec) | 1580 – 2200 | 3000 – 8600 | 4200 – 10000 | 1140 – 2450 |
| Voxel Size (mm) | 0.27 × 0.27 – 1 × 1 | 0.31 × 0.31 – 1 × 1 | 0.86 × 0.86 – 2 × 2 | 0.94 × 0.94 – 2.5 × 2.5 |
| Slice Thickness (mm) | 0.9 – 1.6 | 1.0 – 3.0 | 2.0 – 5.0 | 4.0 – 6.0 |
| Flip Angle (°) | 8 – 180 | 90 – 180 | 90 | 30 – 90 |
| Inversion Time (msec) | 900 – 1100 |  |  |  |
| b Values (sec/mm2) |  |  | 0, 1000 |  |

**Supplementary Figure 1.** The k-means clustering was applied for T2-weighted and contrast-enhanced T1-weighted images to construct structural MRI habitats, and for apparent diffusion coefficient (ADC) and cerebral blood volume (CBV) images to construct physiologic MRI habitats. Enhancing, solid low-enhancing, and nonviable tissue habitats are indicated by red, green, and blue subregions on the structural MRI habitats. Similarly, hypervascular, hypovascular cellular, and nonviable tissue habitats are represented by red, green, and blue subregions on the physiologic MRI habitats.

**Supplementary Figure 2.** Comparison of parameters from structural MRI habitat and physiologic MRI habitats between recurrent/progressed tumor and radiation necrosis. The two asterisks indicates P value <.005.
